# 5’UTR point substitutions and N-terminal truncating mutations of *ANKRD26* in acute myeloid leukemia

**DOI:** 10.1186/s13045-016-0382-y

**Published:** 2017-01-18

**Authors:** Caterina Marconi, Ilaria Canobbio, Valeria Bozzi, Tommaso Pippucci, Giorgia Simonetti, Federica Melazzini, Silvia Angori, Giovanni Martinelli, Giuseppe Saglio, Mauro Torti, Ira Pastan, Marco Seri, Alessandro Pecci

**Affiliations:** 1 Medical Genetics Unit, Department of Medical and Surgical Sciences, University of Bologna, Bologna, Italy; 2 Department of Biology and Biotechnology, Laboratories of Biochemistry, University of Pavia, Pavia, Italy; 3 Department of Internal Medicine, IRCCS Policlinico San Matteo Foundation and University of Pavia, Pavia, Italy; 4 Department of Experimental, Diagnostic and Specialty Medicine, Institute of Hematology “L. and A. Seràgnoli”, University of Bologna, Bologna, Italy; 5 Department of Clinical and Biological Sciences, San Luigi Hospital, University of Turin, Orbassano, Turin Italy; 6 Laboratory of Molecular Biology, Center for Cancer Research, National Cancer Institute, National Institutes of Health, Bethesda, MD USA

**Keywords:** *ANKRD26* gene, Acute myeloid leukemia, Inherited predisposition to leukemia, Inherited thrombocytopenia

## Abstract

Thrombocytopenia 2 (THC2) is an inherited disorder caused by monoallelic single nucleotide substitutions in the 5’UTR of the *ANKRD26* gene. Patients have thrombocytopenia and increased risk of myeloid malignancies, in particular, acute myeloid leukemia (AML). Given the association of variants in the *ANKRD26* 5’UTR with myeloid neoplasms, we investigated whether, and to what extent, mutations in this region contribute to apparently sporadic AML. To this end, we studied 250 consecutive, non-familial, adult AML patients and screened the first exon of *ANKRD26* including the 5’UTR. We found variants in four patients. One patient had the c.−125T>G substitution in the 5’UTR, while three patients carried two different variants in the 5’ end of the *ANKRD26* coding region (c.3G>A or c.105C>G). Review of medical history showed that the patient carrying the c.−125T>G was actually affected by typical but unrecognized THC2, highlighting that some apparently sporadic AML cases represent the evolution of a well-characterized familial predisposition disorder. As regards the c.3G>A and the c.105C>G, we found that both variants result in the synthesis of N-terminal truncated ANKRD26 isoforms, which are stable and functional in cells, in particular, have a strong ability to activate the MAPK/ERK signaling pathway. Moreover, investigation of one patient with the c.3G>A showed that mutation was associated with strong *ANKRD26* overexpression in vivo, which is the proposed mechanism for predisposition to AML in THC2 patients. These data provide evidence that N-terminal ANKRD26 truncating mutations play a potential pathogenetic role in AML. Recognition of AML patients with germline *ANKRD26* pathogenetic variants is mandatory for selection of donors for bone marrow transplantation.

**Electronic supplementary material:**

The online version of this article (doi:10.1186/s13045-016-0382-y) contains supplementary material, which is available to authorized users.

Thrombocytopenia 2 (THC2, MIM 188000) is an autosomal dominant disorder caused by monoallelic single nucleotide substitutions in the 5’UTR of the *ANKRD26* gene [1, 2]. Patients have mild to moderate thrombocytopenia, mild or no bleeding tendency, and increased risk of myeloid malignancies, in particular, acute myeloid leukemia (AML). The analysis of 222 consecutive THC2 patients showed that the incidence of AML, myelodysplastic syndromes, and chronic myelogenous leukemia was significantly higher than expected, with an estimated risk of AML 24-fold increased with respect to the general population [3]. The role of *ANKRD26* in hematopoiesis is poorly understood. A recent investigation indicated that thrombocytopenia of THC2 patients is caused by *ANKRD26* overexpression in megakaryocytes due to defective downregulation by RUNX1 and FLI1, which, in turn, derives from impaired binding of these transcription factors to the mutated 5’UTR [4].

A growing body of evidence indicates that a significant proportion of apparently sporadic, adult-onset AML cases originate from a germline predisposition, which often is not recognized [5, 6]. Given the association of variants in the *ANKRD26* 5’UTR with myeloid neoplasms, we investigated whether, and to what extent, mutations in this region contribute to apparently sporadic AML. To this end, we studied 250 consecutive, non-familial, adult AML patients and screened the first exon of *ANKRD26* including the 5’UTR. Genomic DNA was obtained from peripheral blood at the time of diagnosis.

We found three different variants in four patients, whose clinical features are reported in Additional file 1: Table S1. One patient carried the c.−125T>G substitution in the 5’UTR that was previously reported as responsible for THC2 [2]. Review of personal and family history disclosed that this subject had thrombocytopenia since childhood, and one sister and her son had independently received the diagnosis of THC2 due to the same mutation. We could confirm that the c.−125T>G had a germinal origin (Additional file 1: Table S1). Therefore, this AML case represented the evolution of a typical but unrecognized THC2.

Two patients carried the c.3G>A variant of *ANKRD26* that is predicted to cause the loss of the physiologic start codon (p.Met1?). In both patients, we could analyze the DNA from different tissues (urinary epithelium, saliva, and blood collected in complete remission), which demonstrated the germinal origin of the variant. Finally, one patient had the c.105C>G substitution resulting in the generation of a stop codon at position 35 (p.Tyr35*). We thus investigated the effects of these two variants in the 5’ end of the *ANKRD26* coding region.

In patient 2 carrying the c.3G>A, we could obtain RNA from the whole blood collected at the time of diagnosis. We found that *ANKRD26* mRNA expression was strongly increased (about ninefold changes) in the patient compared with healthy controls (Fig. 1), similarly to what was observed with megakaryocytes and hematopoietic progenitors of THC2 patients [4].

**Fig. 1:**
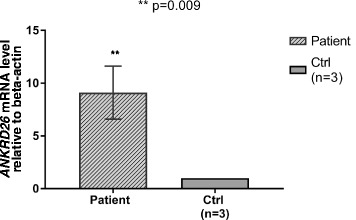
*ANKRD26* is strongly overexpressed in the peripheral blood of one AML patient carrying the c.3G>A variant. Real-time PCR on cDNA from the whole blood showed a significant increase in *ANKRD26* expression in patient 2 with respect to three healthy controls. Data reported represent the mean of the three independent experiments and are expressed as mean ± S.E.M. Statistical analysis was performed by Mann–Whitney non-parametric test

Both the c.3G>A and the c.105C>G are predicted to cause either the complete loss of the ANKRD26 protein or the synthesis of a shorter isoform starting from an ATG downstream the physiologic start codon. To investigate this aspect, we cloned the 5’UTR and either the wild-type (WT) or the mutant *ANKRD26* coding sequences in a 3’ FLAG-tagged vector. After transfection in HeLa cells, ANKRD26 expression was assessed by immunoblotting using the three different antibodies: an anti-FLAG against the C-terminal tag, the JA3 antibody against the N-terminus of human ANKRD26 (residues 1–218), and the SDI antibody recognizing an ANKRD26 internal epitope (residues 289–388) (Additional file 1: Methods). Transfection of WT as well as the mutant constructs resulted in the expression of proteins recognized by the anti-FLAG antibody (Fig. 2a). The WT protein migrated at a molecular mass of about 200 kDa, the predicted mass of full-length ANKRD26, whereas both mutants migrated at a slightly lower mass. Interestingly, the WT ANKRD26 was recognized by all the three antibodies, while the mutant proteins were detected by the anti-FLAG and the SDI, but not the JA3 antibody against the N-terminus (Fig. 2a). These results indicate that the c.3G>A and the c.105C>G have a very similar effect, resulting in the expression of a slightly shorter protein compared to WT ANKRD26, with a truncated N-terminus and a preserved downstream sequence. This picture is consistent with the translation starting from a downstream ATG and then proceeding with a correct and complete reading (Additional file 1: Table S2).

**Fig. 2:**
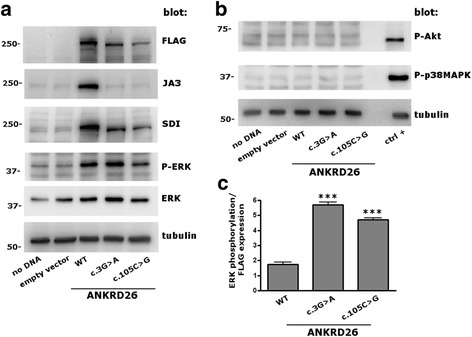
The c.3G>A and c.105C>G variant result in the synthesis of N-terminal truncated proteins that maintain the ability to phosphorylate ERK. ANKRD26-FLAG wild-type (WT) or mutant (c.3G>A or c.105C>G) constructs, or the empty vector, were transfected into HeLa cells. A further control was performed by avoiding DNA loading during transfection (no DNA). **a** Cells were lysed 48 h after transfection and an aliquot of 20 μg of protein was analyzed by immunoblotting. Transfection of both mutant constructs resulted in bands running at a slightly lower molecular mass compared to the WT band, which were recognized by the anti-FLAG and the SDI antibodies, but not by the JA3 antibody. Moreover, transfection of the WT as well as the mutant proteins (but not of the empty vector) induced the phosphorylation of ERK. Tubulin was used as loading control. **b** Transfection of WT or mutant ANKRD26 had no effects on phosphorylation of signaling kinases AKT or p38-MAPK. A lysate of platelets stimulated by 10 μM TRAP was used as positive control (ctrl+). **c** The ability of transfected ANKRD26-FLAG in phosphorylating ERK was measured as the P-ERK/ERK ratio weighted for the amount of FLAG, as determined by densitometric analysis of the respective bands. This value was significantly higher for both mutants compared to WT ANKRD26 (***P < 0.001). Data reported represent the mean of three independent experiments and are reported as mean ± S.E.M. Statistical analysis was performed by Student *t* test

To investigate the stability of the mutant proteins in cells, we blocked the protein synthesis by adding cycloheximide to HeLa cultures 24 h after transfection and measured the kinetics of the subsequent reduction of the amounts of transfected proteins. These experiments showed that both variants presented a similar stability as the WT protein (Additional file 1: Figure S1).

We then investigated whether these mutant proteins maintain their function. The best known functional activity of ANKRD26 is the modulation of different kinase signaling pathways [4, 7], especially the MAPK/ERK pathway. ANKRD26 regulates ERK phosphorylation in mouse embryonic fibroblasts [7]. Hyperactivation of ERK in human megakaryocytes is the mechanism of thrombocytopenia in THC2 and increased ERK signaling at the level of the myeloid progenitors could contribute to predisposition to myeloid malignancies [4]. In HeLa cells, transfection of either WT or mutant ANKRD26 (but not of the empty vector) resulted in a marked phosphorylation of ERK, while it had no effects on some other signal transduction kinases such as AKT or p38MAPK (Fig. 2a, b). The efficiency of exogenous ANKRD26 in phosphorylating ERK was measured as the p-ERK/ERK ratio weighted for FLAG: this value was 2.7- to 3.3-fold higher for the mutants compared with WT ANKRD26 (Fig. 2c). We concluded that the N-truncated ANKRD26 variants do maintain the ability to activate the ERK pathway of the WT protein and could be even more potent ERK activators than the WT ANKRD26.

Interestingly, the c.3G>A and the c.105C>G variants were not present in an in-house cohort of 510 consecutive control individuals of the same geographic origin (Additional file 1: Methods) and resulted in a significantly higher frequency in our cohort of AML patients in comparison to the non-The Cancer Genome Atlas subset of the Exome Aggregation Consortium (exac.broadinstitute.org), with *p* values of 0.012 and 0.032 for the c.3G>A and c.105C>G, respectively.

In summary, the analysis of a large case series showed that variants in the *ANKRD26* 5’UTR are infrequent among non-familial AML patients. However, some apparently sporadic, adult-onset AML cases represent the evolution of an unrecognized THC2. Identification of these cases is imperative especially in patients who are candidates for hematopoietic stem cell (HSC) transplantation from a family donor, in order to avoid the use of HSC from a donor affected by the same inherited disorder. In fact, several reports indicate that the use of HSC from donors with germline mutations predisposing to hematological malignancies resulted in the development of donor-derived leukemia in the recipient and/or poor transplant engraftment [5, 8–10]. Of note, the sister of patient 1 with THC2 developed chronic myelomonocytic leukemia 2 years after the onset of AML in the proband.

Moreover, we observed that mutations in the *ANKRD26* coding sequence resulting in the truncation of the protein N-terminus also have a regulatory effect, causing *ANKRD26* overexpression and thus playing a potential role in AML. In fact, *ANKRD26* overexpression is the proposed pathogenetic mechanism for both thrombocytopenia and predisposition to AML in THC2 patients [4]. Since none of the patients 2–4 presented thrombocytopenia before AML, we suggest that, unlike THC2 mutations, the coding variants described here induce *ANKRD26* overexpression though a mechanism independent of RUNX1/FLI1 interaction with the 5’UTR of the gene and possibly due to increased mRNA stability. In this way, the transcription factors are still able to bind the 5’UTR and downregulate *ANKRD26* in megakaryocytes, thus avoiding thrombocytopenia. Whatever the mechanisms of *ANKRD26* upregulation, we showed that these N-truncated isoforms are stable in cells and have a strong ability to activate the MAPK/ERK pathway. Although further investigation is required, the present data strongly suggest that N-terminal truncating mutations of *ANKRD26* have a potential pathogenetic role in apparently sporadic AML. Since our investigation was restricted to non-familial AML cases, prevalence of *ANKRD26* pathogenetic variants in AML could be greater than we found.

## Additional file

Additional file 1: Table S1. Main clinical and laboratory features of the AML patients with *ANKRD26* mutations identified by the present investigation. **Table S2.** Prediction of translation start codon presence in the *ANKRD26* coding sequence and relative protein size. **Figure S1.** The stability of ANKRD26 mutant proteins is similar to that of the WT counterpart. HeLa cells were transfected by *ANKRD26-FLAG* WT or mutant constructs and cultured in a 12-well plate. Protein synthesis was blocked 24 h after transfection by addition to the cell culture of cycloheximide 100 mM diluted in DMSO. Control conditions were carried out by adding the same amount of DMSO alone. Cells were then lysed just before the addition of cycloheximide or DMSO (time 0) and 8, 24, and 48 h after the addition of cycloheximide or DMSO and analyzed by immunoblotting with anti-FLAG and anti-tubulin antibodies. The histograms show the amount of proteins expressed as FLAG/tubulin ratio and referred to time 0 of each condition. After the addition of cycloheximide, WT ANKRD26 expression decreased to about 60% at 8 h, to 45% at 24 h, and to 20% at 48 h. The expression was significantly lower after cycloheximide treatment compared with DMSO alone at each time point, indicating that protein synthesis was efficiently blocked by cycloheximide (****P* < 0.001; ***P* < 0.01; **P* < 0.05). Overall, mutant and WT proteins showed a similar kinetic of reduction after cycloheximide treatment. Data reported represent the mean of three independent experiments and are reported as mean ± S.E.M. Statistical analysis was performed by Student *t* test. **Methods.** (DOCX 200 kb)

## Abbreviations

AML: Acute myeloid leukemia
HSC: Hematopoietic stem cell
THC2: Thrombocytopenia 2
WT: Wild type
