# Supplementary material for: 5’UTR point substitutions and N-terminal truncating mutations of ANKRD26 in acute myeloid leukemia
Source: J Hematol Oncol. 2017 Jan 18;10:18. doi: 10.1186/s13045-016-0382-y (PMC5242010; doi:10.1186/s13045-016-0382-y)
Supplement: Additional file 1: Table S1. — Main clinical and laboratory features of the AML patients with ANKRD26 mutations identified by the present investigation. Table S2. Prediction of translation start codon presence in the ANKRD26 coding sequence and relative protein size. Figure S1. The stability of ANKRD26 mutant proteins is similar to that of the WT counterpart. HeLa cells were transfected by ANKRD26-FLAG WT or mutant constructs and cultured in a 12-well plate. Protein synthesis was blocked 24 h after transfection by addition to the cell culture of cycloheximide 100 mM diluted in DMSO. Control conditions were carried out by adding the same amount of DMSO alone. Cells were then lysed just before the addition of cycloheximide or DMSO (time 0) and 8, 24, and 48 h after the addition of cycloheximide or DMSO and analyzed by immunoblotting with anti-FLAG and anti-tubulin antibodies. The histograms show the amount of proteins expressed as FLAG/tubulin ratio and referred to time 0 of each condition. After the addition of cycloheximide, WT ANKRD26 expression decreased to about 60% at 8 h, to 45% at 24 h, and to 20% at 48 h. The expression was significantly lower after cycloheximide treatment compared with DMSO alone at each time point, indicating that protein synthesis was efficiently blocked by cycloheximide (***P < 0.001; **P < 0.01; *P < 0.05). Overall, mutant and WT proteins showed a similar kinetic of reduction after cycloheximide treatment. Data reported represent the mean of three independent experiments and are reported as mean ± S.E.M. Statistical analysis was performed by Student t test. Methods. (DOCX 200 kb) [file 13045_2016_382_MOESM1_ESM.docx]

**ADDITIONAL INFORMATION**

**Manuscript:** “**5’UTR POINT SUBSTITUTIONS AND N-TERMINAL TRUNCATING MUTATIONS OF *ANKRD26* IN ACUTE MYELOID LEUKEMIA**” by Marconi et al.

**Additional Table 1.** Main clinical and laboratory features of the AML patients with *ANKRD26* mutations identified by the present investigation.

| **Patient** | **Age/**  **gender** | ***ANKRD26***  **variant** | **Protein effect** | **Mutation origin^1^** | **FAB classification** | **Cytogenetic findings** | **Molecular**  **findings^2^** | **Thrombocytopenia^3^** |
| --- | --- | --- | --- | --- | --- | --- | --- | --- |
| 1 | F/43 | c.-125 T>G | na | germinal | M1 | normal | nd | yes |
| 2 | M/55 | c.3G>A | p.Met1? | germinal | M1 | t(8;21) | no mutations | no |
| 3 | F/26 | c.3G>A | p.Met1? | germinal | M1 | del9 | no mutations | no |
| 4 | F/40 | c.105C>G | p.Tyr35* | nd | M4 | normal | nd | no |

**Notes:** ^1^ = determined by the analysis of DNA obtained from urinary epithelium, saliva, and blood collected during complete remission after conventional chemotherapy. ^2^ = results of the screening for mutations in the following genes: *FLT3*, *NPM1*, *MLL*, *CEPBA*, *NRAS*, *WT*. ^3^ = history of thrombocytopenia before the onset of leukemia and in the remission phase after conventional chemotherapy at clinical review. **Abbreviations:** nd = not determined; na = not applicable.

**Additional Table 2.** Prediction of translation start codon presence in the *ANKRD26* coding sequence and relative protein size.

| **cDNA position** | **Protein position** | **Protein length** | **Protein weight (KDa)** | **NetStart Prediction** | **ATGprediction** | **ORFfinder (NCBI)** |
| --- | --- | --- | --- | --- | --- | --- |
| 1 | 1 | 1710 | 196,45 | Yes | Yes | Yes |
| 235 | 78 | 1632 | 188,08 | Yes | Yes | Yes |
| 352 | 117 | 1593 | 183,76 | No | No | No |
| 580 | 193 | 1517 | 175,47 | No | No | No |
| 844 | 281 | 1429 | 165,66 | No | No | No |
| 1072 | 357 | 1353 | 157,27 | Yes | No | No |
| 1210 | 403 | 1307 | 152,09 | No | No | No |
| 1213 | 404 | 1306 | 151,95 | No | No | No |
| 1387 | 462 | 1248 | 145,65 | No | No | No |
| 1411 | 470 | 1240 | 144,74 | Yes | No | No |
| 1444 | 481 | 1229 | 143,53 | Yes | No | No |
| 1459 | 486 | 1224 | 142,99 | Yes | Yes | No |
| 1504 | 501 | 1209 | 141,17 | Yes | No | No |
| 1537 | 512 | 1198 | 140,08 | Yes | No | No |
| 1672 | 557 | 1153 | 134,8 | Yes | No | No |
| 1834 | 611 | 1099 | 128,84 | No | No | No |
| 2041 | 680 | 1030 | 121,24 | No | No | No |
| 2125 | 708 | 1002 | 118,09 | No | No | No |
| 2149 | 716 | 994 | 117,19 | No | No | No |
| 2266 | 755 | 955 | 112,74 | No | No | No |
| 2524 | 841 | 869 | 102,07 | No | No | No |
| 2617 | 872 | 838 | 98,38 | Yes | No | No |
| 2671 | 890 | 820 | 96,3 | No | No | No |
| 2686 | 895 | 815 | 95,71 | Yes | No | No |
| 2746 | 915 | 795 | 93,39 | No | Yes | No |
| 2767 | 922 | 788 | 92,57 | No | No | No |
| 2959 | 986 | 724 | 85,06 | No | No | No |
| 3142 | 1047 | 663 | 77,88 | No | Yes | No |
| 3322 | 1107 | 603 | 70,83 | No | No | No |
| 3331 | 1110 | 600 | 70,44 | No | No | No |
| 3430 | 1143 | 567 | 66,47 | Yes | No | No |
| 3709 | 1236 | 474 | 55,4 | No | No | No |
| 3859 | 1286 | 424 | 49,55 | Yes | No | No |
| 3925 | 1308 | 402 | 46,93 | No | No | No |
| 4006 | 1335 | 375 | 43,74 | No | No | No |
| 4048 | 1349 | 361 | 42,07 | No | No | No |
| 4105 | 1368 | 342 | 39,8 | No | No | No |
| 4183 | 1394 | 316 | 36,68 | Yes | No | No |
| 4309 | 1436 | 274 | 31,91 | No | No | No |
| 4405 | 1468 | 242 | 28 | No | No | No |
| 4564 | 1521 | 189 | 21,76 | No | No | No |
| 4576 | 1525 | 185 | 21,29 | Yes | No | No |
| 4819 | 1606 | 104 | 11,73 | No | Yes | No |
| 4933 | 1644 | 66 | 7,57 | Yes | No | No |
| 4954 | 1651 | 59 | 6,7 | Yes | No | No |
| 5125 | 1708 | 2 | 0,31 | No | No | No |

**Note:** Prediction was performed with the tools NCBI ORF Finder (ncbi.nlm.nih.gov/gorf/gorf.html), NetStart (cbs.dtu.dk/services/NetStart) and ATGpr (atgpr.dbcls.jp). Interpretation of the results based on the score given by each tool is reported: Yes = presence of a translation start codon; No = absence of a translation start codon; na = not applicable. Protein weight was predicted through the Protein Molecular Weight Calculator online tool (http://www.sciencegateway.org/tools/proteinmw.htm)

Prediction showed the presence of a reliable in-frame start codon downstream to both coding variants described here, in position c.235-237. The predicted molecular weight of the resulting protein (188 KDa) appears consistent with the results of immunoblotting showed in Figure 2.

**Additional Figure 1**

**
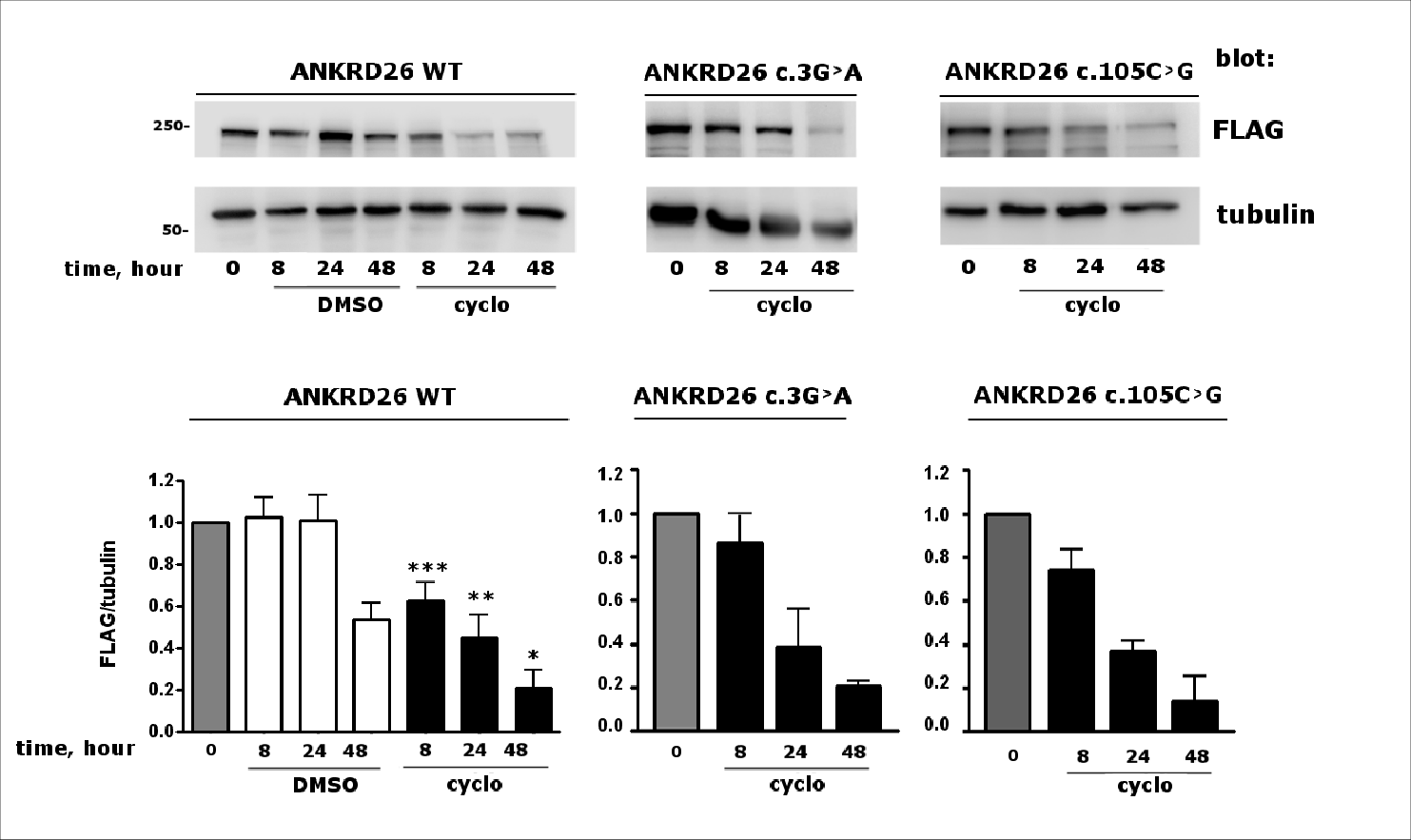
**

**Additional Figure 1. The stability of ANKRD26 mutant proteins is similar to that of the WT counterpart.** HeLa cells were transfected by *ANKRD26-FLAG* WT or mutant constructs and cultured in a 12-well plate. Protein synthesis was blocked 24 hours after transfection by the addition to the cell culture of cycloheximide 100 mM diluted in DMSO. Control conditions were carried out by adding the same amount of DMSO alone. Cells were then lysed just before the addition of cycloheximide or DMSO (time 0) and 8, 24 and 48 hours after the addition of cycloheximide or DMSO and analysed by immunoblotting with anti-FLAG and anti-tubulin antibodies. The histograms show the amount of proteins expressed as FLAG/tubulin ratio and referred to time 0 of each condition. After the addition of cycloheximide, WT ANKRD26 expression decreased to about 60% at 8 hours, to 45% at 24 hours, and to 20% at 48 hours. The expression was significantly lower after cycloheximide treatment compared with DMSO alone at each time point, indicating that protein synthesis was efficiently blocked by cycloheximide (*** *P*<0.001; ***P*<0.01; **P*<0.05). Overall, mutant and WT proteins showed a similar kinetic of reduction after cycloheximide treatment. Data reported represent the mean of three independent experiments and are reported as mean ± S.E.M. Statistical analysis was performed by Student *t* test.

**SUPPLEMENTARY METHODS**

**Patients**

Two hundred and fifty consecutive patients affected with sporadic *de novo* acute myeloid leukemia (AML) were recruited at two different Italian institutions. Genomic DNA was extracted from whole peripheral blood at the time of diagnosis. According to the French-American-British classification, patients were distributed as follows: M0, n=23; M1, n=51; M2, n=45; M3, n=35; M4, n=43; M5, n=53. The study was approved by the Institutional Review Boards of the participating institutions. All subjects provided written informed consent in accordance with the Declaration of Helsinki.

**Screening of *ANKRD26***

Mutational analysis of the exon 1 of *ANKRD26* was performed by PCR amplification using oligonucleotides 1F (5’-CATGGAGCACACTTGACCAC-3’) and 1R (5’-TACTCCAGTGGCACTCAGTC- 3’). PCR and sequence of PCR products were performed by standard methods. The used reference sequence is ENST00000376087.

**RNA extraction and Real Time PCR**

RNA was extracted from whole blood of patient 2 obtained at the time of diagnosis and Reverse Transcription was performed using the High-Capacity cDNA Reverse Transcription Kit (Life Technologies Carlsbad, CA,USA) following manufacturer’s instruction. *ANKRD26* expression in the patient and three healthy controls was evaluated through real time PCR exploiting the UPL technology (Roche, Basel, Switzerland). Data reported represent the fold change of *ANKRD26* expression relative to β-actin levels. Data reported represent the mean of three independent experiments and are expressed as mean ± S.E.M and analyzed with Mann-Whitney non parametric test.

## Plasmids preparation

Full-length human *ANKRD26* coding sequence was isolated from the vector pFN21ASDA1074 (Kazusa DNA Research Institute, Kisarazu, Chiba, Japan) and cloned in the p3XFLAG-CMV-7.1 vector (Sigma-Aldrich, St. Louis, MO, USA) following the manufacturer’s instructions. The human *ANKRD26* 5’UTR was amplified through PCR and inserted in the plasmid upstream of the coding sequence. PCR and Sanger sequencing was performed from bacterial colonies in order to verify the correct orientation and sequence of the plasmid.

Site specific mutagenesis was performed through a circle PCR using the QuikChange XL Site-Directed Mutagenesis Kit (Agilent Technologies, Santa Clara, CA, US) and following the manufacturer’s instructions. Primer sequences are available upon request.

**Cell transfection**

HeLa cells were maintained in RPMI 1640 supplemented with 10% Fetal Bovine Serum and 2mM L-Glutamine (Lonza Group Ltd., Basel, Switzerland). Aliquots of 2.8 µg of *ANKRD26-FLAG* wild-type (WT) or mutant constructs, or the empty vector alone, were transfected into HeLa cells by the Lipofectamine 2000 kit (ThermoFisher Scientific, Waltham, MA, USA) in 12-well plates according to the manufacturer’s instructions. A further control condition was carried out by omitting the DNA during transfection. Cell lysates were prepared 48 hours after transfection as previously reported (1).

**Immunoblotting**

An aliquot of 20 µg of protein was separated on SDS-PAGE (7.5% and 12%). The following antibodies were used: mouse monoclonal M2 against FLAG (Sigma Aldrich); rabbit polyclonal SDI raised against an internal epitope (residues 289-388) of human ANKRD26 (SDIX, Newark, DE, USA, kindly provided by prof. Ira Pastan, National Cancer Institute, National Institutes of Health, Bethesda, MD, USA); mouse monoclonal JA3 raised against the N-terminus (residues 1-218) of human ANKRD26 (kindly provided by Prof. Ira Pastan); mouse monoclonal DM1A against alpha-tubulin and polyclonal antibody against ERK (Santa Cruz Biotechnology, Dallas, TX, USA); rabbit polyclonal antibodies against P-ERK (T202/Y204), P-Akt (S473) and P-p38MAPK (T180/Y182) (Cell Signaling, Danvers, MA, USA). Platelets lysed 3 min after stimulation with 10 µM TRAP4 were used as positive control. Images of reactive bands were acquired using a Chemidoc XRS apparatus (Bio-Rad, Hercules, CA, USA) and quantification of band intensity was performed using the QuantityOne software.

**Assessment of protein stability**

Protein synthesis was blocked by the addition of cycloheximide 100 mM diluted in DMSO to HeLa cultures 24 hours after transfection with WT or mutant *ANKRD26* constructs. Control conditions were carried out by adding the same amounts of DMSO alone. Cells were lysed just before the addition of cycloheximide or DMSO (time 0) and 8, 24 and 48 hours after the addition of cycloheximide or DMSO. The amount of tranfected proteins was then measured by immunoblotting for FLAG and alpha-tubulin as reported above. Data reported represent the mean of three separate experiments and are expressed throughout as mean ± S.E.M and analyzed by *t* test.

**Analysis of Variant Frequencies**

The allelic frequencies of the coding *ANKRD26* variant in the analysed AML cohort was compared with those of two different control groups. One group consisted of 510 consecutive individuals from an internal dataset, having the same geographic origin as the AML cohort and being either healthy or affected with different genetic disorders, but not presenting thrombocytopenia or leukemia. The second control group is non-The Cancer Genome Atlas (TCGA) subset (i.e. excluded the samples from TCGA cohort) of the Exome Aggregation Consortium (ExAC, exac.broadinstitute.org) , including exome data from 53,105 individuals.

For the two variants c.3G>A and c.105C>G, the binomial test was used to calculate the probability of sampling the same number of variant individuals using as prior probability the frequency observed in the ExAC non-TCGA dataset.

**REFERENCE FOR SUPPLEMENTARY METHODS**

1. Necchi V, Balduini A, Noris P, Barozzi S, Sommi P, di Buduo C, Balduini CL, Solcia E, Pecci A. Ubiquitin/proteasome-rich particulate cytoplasmic structures (PaCSs) in the platelets and megakaryocytes of ANKRD26-related thrombocytopenia. Thromb Haemost 2013;109(2):263-71.
